# Peer effects and health impacts of different body cognitive biases in children: micro evidence from China

**DOI:** 10.3389/fpubh.2023.1305795

**Published:** 2024-01-08

**Authors:** Yun Zhang, Xueying Wang, Weiqiang Zhu

**Affiliations:** College of Economics and Management, Nanjing Agricultural University, Nanjing, China

**Keywords:** Children’s body cognitive biases, peer effects, health impacts, diet, sports, body type

## Abstract

**Background:**

In China, children commonly display body cognitive biases, which constitute a significant yet hidden public health issue. These biases potentially jeopardize children’s well-being, hinder the cultivation of human capital, and impede societal progress. However, limited research employs theoretical analysis and econometric testing to investigate the formation of different body cognitive biases among Chinese children and their health impacts.

**Methods:**

Based on a local average network model for theoretical analysis, this study utilizes a sample of 4,289 children from four phases of the China Health and Nutrition Survey (CHNS) conducted from 2004 to 2011. Utilizing Logit and IV Probit models, systematically evaluate the peer effect, heterogeneity of effects, and health impacts of children’s different body cognitive biases.

**Results:**

(1) The peer effect contributes to the development of light- and heavy-body cognitive biases in Chinese children. (2) The heterogeneity analysis shows that the peer effect of body cognitive biases is more significant in rural and female children. (3) The influence of heavy-body cognitive bias is more pronounced in adolescent children. (4) The “eating-activity balance” is disrupted by the two body cognitive biases in children, leading to deviations from normal body type. (5) Specifically, the light-body cognitive bias leads children to intake more and burn fewer calories, increasing their risk of obesity. (6) Conversely, the heavy-body cognitive bias prompts children to intake less and expend more calories, resulting in a higher prevalence of thinness.

**Discussion:**

This study innovates by exploring peer effects on body cognitive biases in Chinese children, elucidating their direction and health implications. While overweight and obesity are recognized as overt health issues, the spread and impact of implicit issues like body cognitive biases should not be overlooked. Nevertheless, the issue is largely neglected in developing countries, such as China, where existing children’s health policies are inadequate in addressing it. Promoting accurate body image perception and understanding of health prevention strategies among children requires adequate attention to peer effects.

## 1 Introduction

Throughout their growth, children face a range of physical health challenges. Traditionally, academics have concentrated on objective physical issues such as overweight, obesity, and malnutrition, yet have not given sufficient attention to body cognitive biases. However, researches indicates that body cognitive biases constitute a significant yet underreported public health issue in China, particularly among young children (1–3). Discrepancies between self-perceived and actual body types represent cognitive biases (4, 5). It and body image dissatisfaction contribute to eating disorders (6), inadequate body management (7), self-worth depreciation (8), social withdrawal (9), reduced self-esteem and autistic depression (10). As children physically and mentally develop, their self-consciousness about their appearance increases (11). Variations in body shape cognition can disrupt growth patterns, posing physical and mental health risks, that threaten the long-term well-being of individuals, families, and nations.

However, the spread and risks of body cognitive biases in Chinese children remain unaddressed in policy-making due to insufficient research. The government primarily promotes resolving obesity and malnutrition in children through various approaches, including reasonable diet, nutritional supplements, and physical exercise.^1^ Instead of studying the emergence of children body cognitive biases, more research is focused on the development of body image dissatisfaction. The causes of body image dissatisfaction can be grouped into four categories: individual social comparisons (12); mass media-driven perception dissemination and socio-cultural transmission (13); peer review (14); parental misperceptions and teaching (15). However, body image dissatisfaction and body cognitive biases are not identical. Body image dissatisfaction can sometimes reflect accurate body condition cognition, whereas body cognitive biases are purely illusory. Furthermore, body image includes various aspects like body form and appearance, which are not equivalent to body type (16). Hence, the formation of body cognitive biases in children necessitates additional research.

Children’s logical reasoning, value judgments, and social cognitive abilities are immature and susceptible to external pressures, such as group reference and peer standards, which may distort their development (17). Based on this point, peer social networks significantly impact children’s health. In terms of physical health, research demonstrated the peer effect on obesity (18), smoking (19), alcohol consumption (20), marijuana use (21), poor fitness (22) and so on. In the field of mental health, investigations into peer effects primarily concentrate on the spread of depression, anxiety, and stress [e.g., (23)]. Nonetheless, most existing research on peer effects rarely explores individual cognition, with only a few studies examining the development of body cognitive biases. It is worth noting that certain literature offers promising research clues regarding the presence of peer effects in children’s body cognitive biases. Some indicates that peer assistance can help improve cognitive and dietary disorders (24). Additionally, normative changes may be an effective mechanism for group psychotherapy (25). More direct clues pointing out misconceptions about peer weight norms or accepting incorrect weight comments from peers may directly influence one’s weight development (26). Peer groups’ cognitive norms about body type may affect individual child’s perception, yet few studies have comprehensively examined this from the peer effects perspective using empirical econometric models for validation.

To effectively address children’s body cognitive biases and adverse consequences, existing research has four main limitations: Firstly, lack of theoretical models and in-depth analysis. The analysis of peer in past studies mainly depends on empirical evidence or simple arguments. However, in other fields of peer effects research, there are existing theoretical models that can be referred to, such as the local average network model (27). Secondly, lack of empirical analysis. The majority of related research employed correlational analysis without rigorous econometrics (1–3, 6–10). Thirdly, lack of attention to children. Previous studies have mainly focused on women and youth groups (4, 28, 29). Given the early onset, widespread prevalence, and enduring consequences of body cognitive bias, children’s groups merit further investigation. Fourthly, lack of research evidence from China. With neither domestic nor international studies providing adequate explanations for the spread of body cognitive biases and health hazards among Chinese children.

Based on the preceding analysis, this study aims to investigate the following questions: Do children’s body cognitive biases exhibit peer effects? If affirmative, are there discrepancies among different characteristic groups such as age, gender, and urban–rural dimensions? Do different types of body cognitive biases have diverse impacts on children’s health? What are the mechanisms behind these varying impacts? Addressing these issues is crucial for boosting Chinese children’s health awareness, reducing early health risks.

This study employs CHNS data to explore the peer effects of children’s body cognitive biases, discerning light- and heavy-body cognitive biases according to subjective and objective body measures. It subsequently addresses endogeneity issues and analyzes the transmission of these biases across various dimensions, along with heterogeneity impacts on healthy body types and core mechanisms. It is structured as follows: Section 2 develops a theoretical framework; Section 3 presents estimation strategies; Section 4 conducts descriptive analysis; Section 5 analyzes empirical results; and Section 6 concludes with discussions.

The contributions include: (1) being the first to investigate the propagation of body cognitive biases among Chinese children; (2) uncovering peer effects and elucidating health consequences across various bias directions; (3) extending the application of peer effects to mental health and cognition.

## 2 Theories and hypotheses

### 2.1 Analysis clues: derived from a simplified local average network model

The local average network model, introduced by Patacchini and Zenou, was developed to study the impact of adolescent conformity on individual criminal (27). This model was later adapted to examine the influence of peer alcohol consumption on individual drinking habits (20). Refer to both studies, we construct a simplified local average network model, offering analytical insights for subsequent theoretical discussions.

Assuming a community has *n* children, *N* = {1,…,*n*} represents a finite set of agents. The *n*-square adjacency matrix *H* represents pairwise relationships among all children in community *h*. In this context, children *i* and *j* connect directly (i.e., best friends), then $h_{ij}$=1; otherwise, $h_{ij}$=0. Considering friendship as a mutual relationship, set $h_{ij}$=$h_{ji}$, $h_{ii}$=0.

$y_{i}\left(h\right)$ denotes the effort level of child *i* in community *h*. It refers to the child’s own body cognition tendency towards a certain body cognitive bias in this community. We define ${\bar{y}}_{i}\left(h\right)$ as the average effort of other children in community, reflecting the average level of a specific body cognitive bias in Eq. 1:


(1) $${\bar{y}}_{i}\left(h\right)=\frac{1}{h_{i}}\sum_{j=1}^{j=n}h_{ij}y_{j}\text{.}$$

We will implicitly omit parameter *h* in subsequent analysis unless further explanation is needed. Child *i* chooses an effort level $y_{i}\geq 0$, yielding utility *u*($y_{i}$,${\bar{y}}_{i}$) with a standard cost/benefit structure. In Eq. 2, *a*, *c* > 0 and $b_{i}$ > 0 for all *i*.


(2) $$u_{i}\left(y_{i},{\bar{y}}_{i}\right)=a+b_{i}y_{i}-py_{i}f-c{\left(y_{i}-{\bar{y}}_{i}\right)}^{2}$$

The benefits of children’s body cognitive bias, represented by $a+b_{i}y_{i}$, increases with his/her effort $y_{i}$. $b_{i}$ denotes individual heterogeneity, reflecting the utility benefit multiplier from child *i*’s body cognitive bias. Within the community, all children have observable utility benefit multipliers associated with various individual and environmental factors, such as gender, age, and community tightness, where $x_{i}^{m}$ represents child *i*’s characteristic variables, explaining differences between *i* and others. $\beta_{m}$ is the parameter:


(3) $$b_{i}\left(x\right)=\sum_{m=1}^{M}\beta_{m}x_{i}^{m}$$

In Eq. 2, the cost of body cognitive bias $py_{i}f$ comprises two parts: $py_{i}$, the expected loss probability, positively correlated with $y_{i}$. *f* embodies the health cost of body cognitive bias, reflected in erroneous body management behaviors and their health consequences. $c{\left(y_{i}-{\bar{y}}_{i}\right)}^{2}$ signifies the psychological utility loss from individual behavior deviation. Assuming children strive for minimal social distance with the reference group, parameter *c* portrays their compliance preference.

The payoff function in a pure strategy compliance game, represented by Eq. 2, has a unique Nash equilibrium, given by Eq. 4:


(4) $$y_{i}^{*}={\bar{y}}_{i}+\left(b_{i}-pf\right)/2c$$

Equation 4 shows child *i*’s equilibrium effort $y_{i}^{*}$ is positively linked to the reference group’s average effort ${\bar{y}}_{i}$, as Eq. 5:


(5) $$\partial y_{i}^{*}/\partial {\bar{y}}_{i}>0$$

Furthermore, $y_{i}^{*}$ increases with utility benefit multiplier $b_{i}$ and decreases with health cost pf for a given ${\bar{y}}_{i}$:


(6) $$\partial y_{i}^{*}/\partial b_{i}>0$$

(7) $$\partial y_{i}^{*}/\partial pf<0$$

### 2.2 Logical expansion: behavioral mechanisms of peer effects in children’s body cognitive biases

Based on the theoretical framework and partial derivatives mentioned above, we further expand the relevant logic.

Equation 5 shows that peer influence impacts children’s body cognitive biases. The Social Cognitive Theory (SCT) suggests that the beliefs of surrounding others are crucial components of an individual’s social environment (30). Discrepancies with group beliefs may cause shame, guilt (31) and lead to negative evaluations and social isolation (32). Children frequently use peer group norms as reference points, altering their behavior to show a desire to imitate and conform. Furthermore, the Social Learning Theory (SLT) suggests that observation and imitation are crucial for behavior acquisition in the face of asymmetric information (33). Children, in a critical stage of mental development, exhibit strong plasticity and face challenges in independent decision-making. They adapt their behaviors by observing and imitating peers in their community, integrating and inferring information. So children in a community internalize physical cognition concepts as group norms (i.e., average body cognitive biases among remaining children in the same community ${\bar{y}}_{i}$). Thereby applying pressure and information on children’s perceptions and behaviors (34). It creates group pressure, spreads group concept info, and fosters individual children’s body cognitive biases $y_{i}^{*}$.

By analyzing Eqs 4, 7, children’s understanding of health risks (pf) related to body cognitive biases may mitigate peer effects. However, as a subjective hidden health issue, children and caregivers often lack adequate health knowledge and risk awareness. For example, caregivers also hold misconceptions about children’s body shapes (35).

*Hypothesis 1*: There are peer effects in children’s body cognitive biases. A stronger group inclination towards a heavy-/light-body cognitive bias prompts children to develop a similar bias.

Equation 6 suggests that increased utility and satisfaction from adopting group perspectives and norms (i.e., $b_{i}$($x_{i}^{m}$) grow) converge children’s body cognition with the group. Equation 3 suggests different $x_{i}^{m}$ offer children heterogeneous utility benefit multipliers $b_{i}\left(m\right)$. It allows for a thorough exploration of peer effects of body cognitive biases in various dimensions.

Firstly, the dimension of community characteristics. Children from various communities interact with diverse social networks, leading to peer effects. Social networks’ structural features include number, mobility, and connectedness (36). An increase in the number of network members enhances density, facilitating more information and behavioral exchanges within groups. Stronger interpersonal linkages amplify the influence of group values and norms on individual behavior. In contrast, urban communities have fewer extensive blood and kinship ties, daily interactions, and communication compared to rural communities. According to Eq. 3, rural communities in China demonstrate stable membership, cohesive living conditions, and heightened child-to-child interactions. Social norms and attitudes have a more significant impact on rural children (i.e., $b_{i,\mathrm{rural}}>b_{i,\mathrm{urban}}$).

*Hypothesis 2*: Rural children are more susceptible to the influence of peer effects on body cognitive biases compared to their urban counterparts.

Secondly, the dimension of gender characteristics. Gender awareness among children is escalating (37). Local culture can influence ideal body shape stereotypes (38). Traditional Chinese culture and aesthetics shape societal expectations and gender norms concerning body types, prioritizing masculinity and strength for men, and slimness and softness for women (39). According to the Gender Schema Theory (40), individuals’ assumed roles or identities correspond to particular social expectations, and any deviation may lead to public pressure or losses. Children’s peer environments demonstrate differences in biological and social genders. So male children benefit more from adhering to the group’s light-weight body cognitive bias norms, the situation is reversed for female children (i.e., $b_{i,\mathrm{boy},\mathrm{light}-\mathrm{body cognitive bias}}>b_{i,\mathrm{boy},\mathrm{heavy}-\mathrm{body cognitive bias}}$, $b_{i,\mathrm{girl},\mathrm{heavy}-\mathrm{body cognitive bias}}>b_{i,\mathrm{girl},\mathrm{light}-\mathrm{body cognitive bias}}$). Furthermore, women are more vulnerable to experiencing beauty premiums and ugly penalties in learning, employment, and marriage (41, 42), and they are at a higher risk of experiencing body shape anxiety. Considering these issues, females are more likely to engage in conversations about beauty and body type (29). Group body cognitions have a more substantial impact (i.e., $b_{i,\mathrm{girl}}>b_{i,\mathrm{boy}}$).

*Hypothesis 3*: Light-body cognitive biases have a stronger peer influence on male children, while heavy-body cognitive biases are more influential on female children. In general, females are more prone to peer influence.

Finally, the dimension of age characteristics. Adolescents tend to rebel, question adult authority, and challenge the views and guidance of parents and teachers (43). Adolescent children exhibit increased intimacy with their peers, are more likely to derive a sense of belonging and identity from peer groups (33), and are more susceptible to the influence of group physical concepts. Adolescent children’s self-assessments of their internal and external appearance fluctuate (34), and they are concerned with mainstream social aesthetics. In today’s society, which values facial attractiveness and body shape, adolescents are more prone to heavy-body cognitive biases. Given these concerns, $b_{i,\mathrm{adolescence}}>b_{i,non-\mathrm{adolescence}},b_{i,\mathrm{adolescence},\mathrm{heavy}-\mathrm{body cognitive bias}}$ $>b_{i,\mathrm{adolescence},\mathrm{light}-\mathrm{body cognitive bias}}$.

*Hypothesis 4*: Adolescent children are more affected by peer effects regarding body cognitive biases than non-adolescents, particularly the heavy-body cognitive bias.

## 3 Methodology and variables

### 3.1 Sample

The China Health and Nutrition Survey (CHNS) is an international collaborative effort conducted by the University of North Carolina at Chapel Hill and the Chinese Center for Disease Control and Prevention.^2^ This survey has been carried out in 1989, 1993, 1997, 2000, 2004, 2006, 2009, 2011, and 2015. The multistage random cluster sampling method employed in the survey, which is based on distinct income levels (high, medium, and low) and weighted sampling, involves the following steps: after randomly selecting four counties and two cities within each province, the CHNS randomly identifies villages and towns in each county, as well as urban and suburban regions in each city. Subsequently, 20 households are selected from each of these communities. The survey covers provinces such as Liaoning, Heilongjiang, Jiangsu, Shandong, Henan, Hubei, Hunan, Guizhou, and Guangxi. These locations can be found at: https://www.cpc.unc.edu/projects/china/about/proj_desc/chinamap. In 2011, Beijing, Shanghai, and Chongqing were incorporated into the study. The control variables in this paper encompass parents’ knowledge of dietary guidelines. As the indicators for this variable were only available from 2004 onwards, and we lacked comprehensive access to the CHNS 2015 data, we examined four periods of mixed cross-sectional data spanning 2004 to 2011, covering children aged 6 to 17 years. Samples with missing crucial data, logistic anomalies, or severe physical limitations (e.g., blindness, arm function loss, leg function loss) were excluded, resulting in 4,289 valid observations. Due to variable availability, the sample size was reduced when estimating and detecting the health effects of body cognitive biases using instrumental factors.

Although not the latest, CHNS2004-2011 remains a valuable resource for studying peer effects on children’s body cognitive biases. The CHNS is the only public dataset currently recording subjective and objective body data for Chinese children. The detailed personal, familial, and community characteristics in the CHNS dataset aid in conducting quantitative empirical tests on peer effects. As discussed, the Chinese government’s lack of policy addressing children’s body cognitive biases justifies the concern over its presence in modern China.

### 3.2 Empirical strategy

#### 3.2.1 Validate peer effects

To investigate peer effects on children’s body cognitive biases, a logit model was constructed with group body cognitive biases as the core independent variables, and children’s body cognitive bias as the dependent variable, using a heteroscedasticity-robust standard error. Referring to Lee (44), the econometric model is specified as Eq. 8 and Eq. 9.


(8) $${y_{i}}_{ct}=\alpha_{0}+\alpha_{1}\bar{y_{-ict}}+\alpha_{2}{X_{i}}_{ct}+\alpha_{3}{Z_{i}}_{ct}+{\varepsilon_{i}}_{ct}$$

(9) $$\bar{y_{-ict}}=\left(\sum {y_{i}}_{ct}-{y_{i}}_{ct}\right)/\left(N_{ct}-1\right)$$

where ${y_{i}}_{ct}$ denotes that child *i* in community *c* exhibits a light- or heavy-body cognitive bias during period *t*; $\bar{y_{-ict}}$ represents the mean of children’s body cognition biases (light- or heavy-body cognitive biases) in period *t*, except for child *i*, in the community *c*; $X_{ict}$ represents the child and familial characteristic factors; ${Z_{i}}_{ct}$ represents the characteristics of child’s community; $\varepsilon_{ict}$ serves as the model perturbation term. $N_{ct}$ denotes the total number of children residing in community *c* during period *t*.

#### 3.2.2 Relieve endogeneity

As Manski (45) stated, the study of peer effects often encounters the common issue of reflexivity. Synchronous cognition transmission may result in reverse causality in assessing peer effects. To address it, we employ instrumental variables and the IV-Probit econometric model as Eq. 10–12.^3^


(10) $$y_{1,ict}^{*}=\alpha x_{ict}+\phi y_{2,ict}+\mu_{ict}$$

(11) $$y_{2,ict}=\gamma x_{ict}+\eta X_{ict}+\nu_{ict}$$

(12) $$y_{1,ict}=1\left(y_{1,ict}^{*}>0\right)$$

The observable dummy variable $y_{1,ict}$ indicates whether the child has a light- or heavy-body cognitive bias; $y_{1,ict}^{*}$ represents the corresponding latent variable. Meanwhile, $y_{2,ict}$ serves as the endogenous independent variable; $\mu_{ict}$, $\nu_{ict}$ denote the perturbation terms.

#### 3.2.3 Validate health impacts

To examine health impacts, a logit model was constructed with “*Body Type Deviation*” as the dependent variable (including deviations from normal body type, tendencies toward overweight, and tendencies toward thinness). The child’s body cognitive bias serves as the core independent variable. Given the common factors influencing health related issues, the control variables align with the baseline model as Eq. 13.


(13) $$\delta_{ict}=\beta_{0}+\beta_{1}y_{ict}+\beta_{2}X_{ict}+\beta_{3}Z_{ict}+\tau_{ict}$$

When $\delta_{ict}$ represents whether the body type deviates from the norm, the two types of children’s body cognitive biases $y_{ict}$ act as core independent variables. When $\delta_{ict}$ suggests a tendency towards overweight, the light-body cognitive bias act it; and when it suggests a tendency towards thinness, the heavy-body perception bias act it.

### 3.3 Variables

#### 3.3.1 Dependent variable: $y_{ict}$

$y_{ict}$ represents the light- or heavy-body cognitive bias of child *i* in community *c* during period *t*. Value 1 indicates the presence of the body cognitive bias, while 0 signifies its absence. The CHNS dataset comprises objective body indicators such as weight and height. And the survey inquires “Do you think you are now underweight, normal or overweight?” to ascertain the child’s body cognitive type.^4^

The 2016 Dietary Guidelines for Chinese Residents^5^ include a children’s BMI metric in its appendix.^6^ BMI intervals were defined based on the age and gender of children to define overweight, normal, and underweight. We utilized children’s BMI and the classification criteria from the guidelines to objectively determine body types.

Referring to existing literature (4, 5), the following situations belong to light-body cognitive bias: children who have a normal body type but believe they are underweight; children who are overweight but believe they are underweight or normal. The following situations belong to heavy-body cognitive bias: children who have a normal body type but believe themselves as overweight; thin body type but believe themselves as normal, or overweight.

#### 3.3.2 Core independent variable: $\bar{y_{-ict}}$

The core independent variable $\bar{y_{-ict}}$ represents the mean level of light- or heavy-body cognitive biases among all children in community *c* and period *t*, excluding child *i*. It’s a [0, 1] continuous variable. Value 0 indicates that no children in the group exhibit body cognitive biases, while value 1 signifies that all children do.

#### 3.3.3 Control variables

Manski (45) highlighted the challenges in accurately identifying peer effects, citing interference from correlated effects and exogenous effects. The correlated effect, in this context, pertains to the co-relation in body cognitive biases among individuals within a community, potentially induced by unobservable yet similar critical factors among these individuals. The exogenous effect denotes the influence of shared environmental factors on the body cognitive biases of diverse individuals.

To overcome interference factors and precisely identify peer effects, we select the following control variables: firstly, in terms of correlated effects, owing to the random assignment of individuals within the same community in the CHNS data, rather than being a consequence of children’s self-selection, this approach effectively mitigates the correlation effects arising from self-selection biases (18). Secondly, in terms of exogenous effects, reviewing Huang et al. (46), we refined the relevant control variables in order to isolate exogenous effects from endogenous effects. Considering children’s height and body size, as well as social aesthetics, vary by province and evolve over time, fixed effects for province and survey year were introduced to control for unobserved group effects (47). To manage the impact of the community environment and gathering places for children, we incorporated three variables: the community food environment (have fast food restaurants or not), leisure activity environment (have recreational activity venues or not), and internet entertainment environment (have Internet cafes or not). Additionally, we regulated for the children’s individual characteristics (gender, age, education level, etc.) and family attributes (number of siblings, average parental age, average parental BMI, highest parental education level, parental living conditions, annual family income, and parental awareness of dietary guidelines, etc.), as well as family residency.^7^

## 4 Descriptive statistics

The descriptive statistics for the variables are presented in Table 1. The sample showed that over half of the Chinese children surveyed face body cognitive bias issues, 38% of participants had a heavy-body cognitive bias, while 13% had a light-body cognitive bias. Given the sample’s wide geographical distribution and lack of policy attention to this issue, it is possible that the current situation with body cognitive bias among Chinese children is more severe. The sample had a nearly 1:1 gender ratio and a reasonable distribution. The age range of 6–17 years old represents the majority of children still in education, reflecting the reality of the situation.

**Table 1:** Descriptive analysis of main variables (*N* = 4,289).

Variables	Variable assignment	Mean	SD	Min	Max
***Dependent variables***					
Heavy-body cognitive bias	1 = yes, 0 = no	0.38	0.49	0	1
Light-body cognitive bias	1 = yes, 0 = no	0.13	0.33	0	1
***Core independent variables***					
Group heavy-body cognitive bias	0–1	0.37	0.23	0	1
Group light-body cognitive bias	0–1	0.12	0.16	0	1
***Control variables***					
Child’s gender	1 = male, 0 = female	0.53	0.50	0	1
Child’s age	years old	11.55	3.25	6	17
Child’s education level	codes, 0–5	1.06	0.98	0	5
Child’s school attending	1 = yes, 0 = no	0.94	0.25	0	1
Child’s siblings	individual	0.32	0.57	0	3
Parents’ highest education level	codes, 0–6	2.46	1.21	0	6
Parents’ average age	years old	38.67	4.98	26	54.5
Parents’ average BMI	kg/m^2^	23.12	2.52	15.82	33.85
Father’s home situation	1 = home, 0 = outside	0.14	0.35	0	1
Mother’s home situation	1 = home, 0 = outside	0.02	0.15	0	1
Parents’ knowledge of dietary guidelines	1 = know, 0 = not know	0.20	0.40	0	1
Annual household income	million yuan/year	2.71	2.88	0	14.6
Community’s leisure activities place	1 = yes, 0 = no	0.21	0.41	0	1
Community’s fast food restaurants	1 = yes, 0 = no	0.22	0.42	0	1
Community’s Internet Cafe	1 = yes, 0 = no	0.44	0.50	0	1
Family residences	1 = urban, 0 = rural	0.31	0.46	0	1
Child’s average daily caloric intake^a^	kcal/day	1741.53	591.41	657.79	3526.29
Child’s sports activities participation^b^	1 = yes, 0 = no	0.85	0.36	0	1

Codes: 0 illiterate, 1 elementary school, 2 junior high school, 3 high school, 4 polytechnic school, 5 university 6 master and above.

a Due to the absence of this indicator in some samples, the actual number of regression samples in identifying the health effect mechanism of form cognitive bias was 4166.

b Similarly, the actual sample size of the latter regression is 3,940 due to the absence of this indicator in some samples.

A comparison was drawn between normal-shaped children and those who were thin, overweight, and obese to examine the distribution of body cognition. Table 2 reveals that over 20% of normal-shaped children perceived their body shapes as abnormal, while more than 60% of children with abnormal body shapes believed their shapes were normal. These findings suggest that body cognitive biases are common among Chinese children, especially those with atypical body types.

**Table 2:** Distribution of body cognition among children with different body types.

Body cognition	Normal body type		Abnormal body type	
	Number of samples	Proportion %	Number of samples	Proportion %
Light-	180	11.52	730	26.77
Normal-	1,203	77.02	1,697	62.23
Heavy-	179	11.46	300	11.00
Total	1,562	100	2,727	100

Compiled from CHNS data (2004–2011).

Table 3 reveals that children with light-body cognitive biases exhibit a higher group light-body cognitive bias level (0.17) compared to those without such biases (0.11), as indicated in column 4. Similarly, column 3 shows that children with heavy-body biases have a higher group heavy-body cognitive bias level (0.41) than those without heavy-body cognitive biases (0.34). Column 5 reveals that 33% of children with light-body biases and 11% of those with heavy-body biases have normal body shapes. The proportion of normal body types in both groups is lower than in children without cognitive biases.

**Table 3:** Comparison of group bias, gender distribution, and body size deviation for body cognitive biases.

Child’s body cognitive biases	Presence	Group heavy-body cognitive bias level	Group light-body cognitive bias level	Proportion of normal body type
Light-	No	0.38	0.11	0.37
	Yes	0.31	0.17	0.33
Heavy-	No	0.34	0.13	0.52
	Yes	0.41	0.10	0.11

Compiled from CHNS data (2004–2011).

## 5 Results

### 5.1 Baseline results

Table 4 presents the Logit marginal regression results of peer effects for both body cognitive biases. Columns 1 and 3 only include core independent variables and the dependent variable. Columns 2 and 4 further control for individual, family, and community characteristics. Column 2 shows a 14.2% increase in child’s light-body cognitive bias likelihood for each 1-unit increase in the community’s group light-body cognitive biases (*p* < 0.01). Similarly, Column 4 indicates a 13.8% surge in heavy-body cognitive bias probability for every 1-unit increase in the community’s group heavy-body cognitive biases (*p* < 0.01). Hypothesis 1 is not rejected. The control variables estimations were consistent with the known empirical experiences.

**Table 4:** Peer effects for the two types of body cognitive biases.

Variables	Light-body cognitive bias		Heavy-body cognitive bias	
	(1)	(2)	(3)	(4)
Group light-body cognitive bias	0.233***	0.142***		
	(8.443)	(4.842)		
Group heavy-body cognitive bias			0.293***	0.138***
			(9.478)	(4.347)
Child’s gender		0.049***		−0.005
		(4.804)		(−0.388)
Child’s age		−0.008**		−0.028***
		(−2.316)		(−5.404)
Child’s education level		0.013		−0.034**
		(1.165)		(−1.991)
Child’s school attending		−0.047**		0.132***
		(−2.328)		(3.763)
Child’s siblings		−0.012		0.011
		(−1.085)		(0.861)
Parents’ highest education level		−0.001		−0.002
		(−0.137)		(−0.218)
Parents’ average age		−0.000		−0.002
		(−0.247)		(−0.940)
Parents’ average BMI		0.004*		−0.013***
		(1.898)		(−4.390)
Father’s home situation		0.011		0.011
		(0.734)		(0.535)
Mother’s home situation		−0.025		−0.000
		(−0.693)		(−0.005)
Parents’ knowledge of dietary guidelines		−0.013		−0.017
		(−0.948)		(−0.857)
Annual household income		0.003		−0.007**
		(1.612)		(−2.510)
Community’s leisure activities place		0.012		−0.015
		(0.928)		(−0.779)
Community’s fast food restaurants		0.024*		−0.010
		(1.772)		(−0.526)
Community’s internet cafe		0.003		0.002
		(0.267)		(0.114)
Family residences		0.019*		−0.045***
		(1.680)		(−2.688)
Year fixed effects	No	Yes	No	Yes
Provincial fixed effects	No	Yes	No	Yes
*N*	4,289	4,289	4,289	4,289

* *p* < 0.10, ** *p* < 0.05, *** *p* < 0.01; *z*-values under robust standard errors are in parentheses.

### 5.2 Robustness tests

The robustness of the baseline regression is assessed by utilizing joint fixed effects, excluding specific samples, considering survey months, and applying “dummy peer” counterfactuals. Table 5 presents the test results, supporting the conclusion of baseline regression.

**Figure 1:**
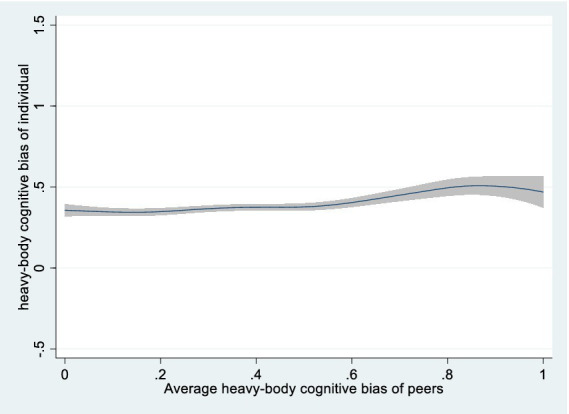
Semiparametric estimation of average peer effects on heavy-body cognitive bias (nonparametric part).

**Figure 2:**
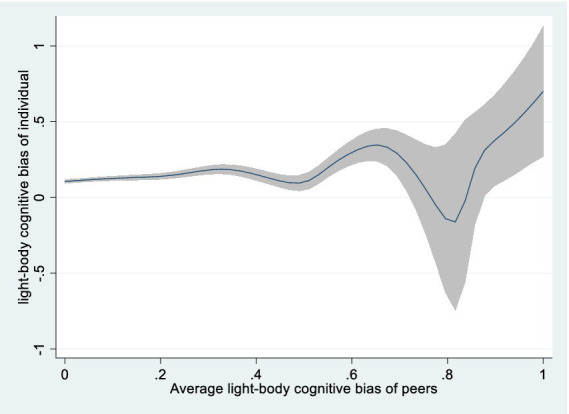
Semiparametric estimation of average peer effects on light-body cognitive bias (nonparametric part).

1. Using joint fixed effects. Although province and year fixed effects were considered in the baseline regression, district-level factors varying by year might have been overlooked. To account for regional trend effects, we incorporated joint year-provincial fixed effects. Columns 1 and 2 indicate that the child’s light-body cognitive bias likelihood increases by 10.8% (*p* < 0.01) and heavy-body cognitive bias probability surges by 11% (*p* < 0.01) for each 1-unit increase in the community’s corresponding group cognitive biases.
2. Excluding non-school sample. School-aged children (6–17 years) are typically found in a school setting. However, some are no longer in school due to personal, familial, and institutional factors, leading to increased community time and peer pressure. On the other hand, these may experience heightened social isolation and detachment from peers. To minimize potential impacts, non-enrolled children are excluded. Columns 3 and 4 demonstrate that light-body cognitive bias likelihood increases by 14.5% (*p* < 0.01) and heavy-body cognitive bias probability surges by 12.8% (*p* < 0.01) with each 1-unit increase in the community’s corresponding group cognitive biases.
3. Excluding recent sickness sample. Children experiencing health shocks with increased risk perception may reevaluate their body type and adjust body cognition (48). To mitigate the impact of recent health risk shocks, unwell or injured children within four weeks prior to the interview were excluded. Columns 5 and 6 support the baseline findings, light-body cognitive bias likelihood increases by 14.3% (*p* < 0.01) and heavy-body cognitive bias probability surges by 13.8% (*p* < 0.01) per 1-unit increase in the community’s corresponding group cognitive biases.
4. Controlling survey month. Cold environments result in heavier, warmer clothing, decreasing peer visibility and peer effects, whereas warm weather prompts lighter attire. Consequently, the baseline regression incorporates survey month. Columns 7 and 8 demonstrate that light-body cognitive bias likelihood increases by 13.3% (*p* < 0.01) and heavy-body cognitive bias probability rises by 13.5% (*p* < 0.01) with each 1-unit increase in the community’s corresponding group cognitive biases.
5. Counterfactual test. To eliminate county-level correlation effects, we adopted Li′s study (49), where children from other communities in the same county were randomly sampled with put-back and matched to target community children as “dummy peers.” Light- and heavy-body cognitive biases were then recaptured. The non-significant “dummy peer” effect in columns 9 and 10 indicated no county-level correlation impact.
6. Semiparametric method. We estimated a semiparametric partially linear model (PLM), using Robinson’s double residual method (50). Figures 1, 2 reveal a positive relation between group body cognitive bias levels and individual body cognitive bias.

**Table 5:** Robustness test results.

Panel A					
Variables	(1) Light-	(2) Heavy-	(3) Light-	(4) Heavy-	(5) Light-
Group light-body cognitive bias	0.108***		0.145***		0.143***
	(3.722)		(4.885)		(4.708)
Group heavy-body cognitive bias		0.110***		0.128***	
		(3.435)		(3.855)	
Control variables	Yes	Yes	Yes	Yes	Yes
Year fixed effects	Yes	Yes	Yes	Yes	Yes
Provincial fixed effects	Yes	Yes	Yes	Yes	Yes
Year-province fixed effects	Yes	Yes	No	No	No
N	4,289	4,289	4,011	4,011	3,901
Panel B					
Variables	(6) Heavy-	(7) Light-	(8) Heavy-	(9) Light-	(10) Heavy-
Group light-body cognitive bias		0.133***			
		(4.504)			
Group heavy-body cognitive bias	0.138***		0.135***		
	(4.166)		(4.252)		
Group light-body cognitive bias I				0.009	
				(0.259)	
Group heavy-body cognitive bias I					−0.003
					(−0.097)
Control variables	Yes	Yes	Yes	Yes	Yes
Year fixed effects	Yes	Yes	Yes	Yes	Yes
Provincial fixed effects	Yes	Yes	Yes	Yes	Yes
Year-province fixed effects	No	No	No	No	No
August		0.040	−0.035		
		(0.872)	(−0.592)		
September		0.052	−0.029		
		(1.158)	(−0.497)		
October		0.070	−0.044		
		(1.548)	(−0.738)		
November		0.083*	−0.048		
		(1.819)	(−0.801)		
December		0.115**	−0.058		
		(2.240)	(−0.863)		
*N*	3,901	4,289	4,289	4,289	4,289

*p < 0.10, **p < 0.05, ***p < 0.01; z-values under robust standard errors are in parentheses.

### 5.3 Endogenous issues

When assessing peer effects, synchronous perception transmission can create reverse causal endogeneity, biasing coefficient estimates. Referring to Sund (51), we use the lagged level of group body cognitive bias as an instrumental variable and using IV Probit model to estimate peer effects. Given that CHNS survey intervals are 2–3 years and community composition remains stable in the short term, cognitive biases in the lag period are closely related to the current period, unaffected by the current period’s perturbation term, thus representing a suitable instrumental variable. However, the CHNS data contains samples with tracking failures, reducing the sample size. Table 6 column 1 and 3 reveals the first-stage F-test values for heavy- and light-body cognitive biases are 27.768 and 24.561, respectively, suggesting no weak instrumental variable issue. Column 2 and 4 show *p*-values from the Wald test of exogeneity for peer effects of both body cognitive biases, with original variables considered endogenous, are 0.004 (*p* < 0.01) and 0.039 (*p* < 0.05). Although the IV-Probit two-stage estimation results indicate that the baseline conclusions remain valid (*p* < 0.01).

**Table 6:** IV-Probit two-stage estimation results.

Variables	Phase I	Phase II	Phase I	Phase II
	Group heavy-body cognitive bias	Heavy-body cognitive bias	Group light-body cognitive bias	Light-body cognitive bias
	(1)	(2)	(3)	(4)
Group heavy-body cognitive bias (lagged one period)	0.141***			
	(5.269)			
Group heavy-body cognitive bias		3.711***		
		(2.612)		
Group light-body cognitive bias (lagged one period)			0.139***	
			(4.956)	
Group light-body cognitive bias				5.346**
				(2.158)
Control variables	Yes	Yes	Yes	Yes
Year fixed effects	Yes	Yes	Yes	Yes
Provincial fixed effects	Yes	Yes	Yes	Yes
Weak IV test *F*-value	27.768		24.561	
Exogenous Wald test *p*-value		0.004		0.039
*N*	1,344	1,344	1,344	1,344

*p < 0.10, **p < 0.05, ***p < 0.01; *t*-values under robust standard errors are estimated in parentheses in the first stage, and *z*-values in the second stage.

### 5.4 Heterogeneity analysis

1. Urban–rural heterogeneity. Children were grouped and regressed by community type to compare peer effects of two body cognitive biases in urban and rural areas. Table 7 shows that light- and heavy-body cognitive biases have significant peer effects in rural areas but not in urban (*p* < 0.01). Hypothesis 2 is valid. According to prior theory, urban and rural communities vary in individual stability, ties density, and daily communication regularity, influencing potential and strength of peer effects.
2. Gender heterogeneity. Group regressions were used to examine gender heterogeneity in the peer effect of two body cognitive biases. Table 8 columns 1 and 3 show that male children are more affected by light-body cognitive biases than females, with a higher coefficient (0.119 > 0.077) and statistical significance. The comparative analysis of columns 1 and 2, as well as columns 3 and 4, reveals that the peer effect is stronger for both heavy- and light-body cognitive biases in female children than males (0.212 > 0.077, 0.160 > 0.119). Hypothesis 3 is valid.
3. Age heterogeneity. The World Health Organization (WHO) categorizes children under 10 as non-adolescents, and those 10 and older as adolescents.^8^ Table 9 columns 1 and 2 show that adolescents are more affected by heavy-body cognitive bias than light-body bias (0.144 > 0.131). However, columns 3 and 4 indicate the peer effect of light-body cognitive bias was weaker in adolescents (0.110 < 0.235). Hypothesis 4 is partially valid. This could be attributed to societal aesthetics, emphasizing thinness, significantly influencing children’s body perceptions.

**Table 7:** Urban–rural grouping regression results.

Variables	Heavy-body cognitive bias		Light-body cognitive bias	
	(1) Urban	(2) Rural	(3) Urban	(4) Rural
Group heavy-body cognitive bias	−0.005	0.163***		
	(−0.081)	(4.210)		
Group light-body cognitive bias			−0.051	0.160***
			(−0.869)	(5.011)
Control variables	Yes	Yes	Yes	Yes
Year fixed effects	Yes	Yes	Yes	Yes
Provincial fixed effects	Yes	Yes	Yes	Yes
*N*	1,323	2,966	1,323	2,966

*p < 0.10, **p < 0.05, ***p < 0.01; *z*-values under robust standard errors are in parentheses.

**Table 8:** Gender grouping regression results.

Variables	Heavy-body cognitive bias		Light-body cognitive bias	
	(1) Male	(2) Female	(3) Male	(4) Female
Group heavy-body cognitive bias	0.077*	0.212***		
	(1.805)	(4.517)		
Group light-body cognitive bias			0.119***	0.160***
			(2.689)	(4.398)
Control variables	Yes	Yes	Yes	Yes
Year fixed effects	Yes	Yes	Yes	Yes
Provincial fixed effects	Yes	Yes	Yes	Yes
*N*	2,275	2014	2,275	2014

*p < 0.10, **p < 0.05, ***p < 0.01; *z*-values under robust standard errors are in parentheses.

**Table 9:** Age grouping regression results.

Variables	Heavy-body cognitive bias		Light-body cognitive bias	
	(1) Adolescence	(2) Non-adolescence	(3) Adolescence	(4) Non-adolescence
Group heavy-body cognitive bias	0.144***	0.131**		
	(3.904)	(2.144)		
Group light-body cognitive bias			0.110***	0.235***
			(3.221)	(3.972)
Control variables	Yes	Yes	Yes	Yes
Year fixed effects	Yes	Yes	Yes	Yes
Provincial fixed effects	Yes	Yes	Yes	Yes
*N*	2,976	1,313	2,976	1,286

*p < 0.10, **p < 0.05, ***p < 0.01; *z*-values under robust standard errors are in parentheses.

### 5.5 Further analysis: the health implications of two body cognitive biases

Children’s body cognitive biases reduce utility. To counteract this, they use various methods, including food, exercise, and medical techniques, to change body shape (6, 7, 28). Nevertheless, it’s uncertain whether Chinese children develop incorrect weight management behaviors due to body cognitive biases. We aim to explore the relationship between body cognitive biases and body size deviation in children.

#### 5.5.1 Whether children’s body cognitive bias affects body type

We first examined the relationship between two body cognitive bias and body type deviation, using “whether it deviated from the normal body type” as the dependent variable. Table 10 columns 1 and 2 show that deviation from normal body type increases regardless of form biases. Then, we examined the effects of two body cognitive biases on body type deviation by using “whether or not it tends to be overweight” and “whether or not it tends to be thin” respectively. Columns 3 and 4 show that light-body cognitive bias increases the likelihood of being overweight-obese by 25.7% (*p* < 0.01), and heavy-body cognitive bias increases the likelihood of being thin by 36.7% (*p* < 0.01).

**Table 10:** Two types of body cognitive bias and children’s body type deviation.

Variables	(1) Normal body	(2) Normal body	(3) Overweight and above	(4) Thin
Light-body cognitive bias	−0.030*		0.257***	
	(−1.923)		(37.362)	
Heavy-body cognitive bias		−0.246***		0.367***
		(−21.659)		(49.830)
Control variables	Yes	Yes	Yes	Yes
Year fixed effects	Yes	Yes	Yes	Yes
Provincial fixed effects	Yes	Yes	Yes	Yes
*N*	4,289	4,289	4,289	4,289

*p < 0.10, **p < 0.05, ***p < 0.01; *z*-values under robust standard errors are in parentheses.

#### 5.5.2 How children’s body cognitive bias affects body type

Some literature indicates that body cognition can cause eating disorders (EDs) (6). However, many of them focus on specific foods (1). Changes in calorie intake significantly influence obesity or body leanness, yet the existing literature on body cognition lacks direct evidence. Furthermore, when calorie intake and expenditure change together, it’s not certain to cause obesity or weight loss. The literature lacks comprehensive studies on the effects of body cognitive biases on calorie intake and expenditure through the lens of eating and exercise balance.

From “eating-activity balance,” we identified how body cognitive biases affect body shape change. Using CHNS data on respondents’ average daily calorie intake over the previous 3 days and “Child’s average daily caloric intake” as the dependent variable. Left-handed Tobit regression model marginal estimation results are in Table 11 columns 1 and 2. CHNS data also counted children’s participation in martial arts, gymnastics, dance, and ball games. Participation in one of these sports was considered sports participation, “child’s sports participation” was the dependent variable. Logit regression model marginal estimation results are in columns 3 and 4. Children with a heavy-body cognitive bias reduce calorie intake 37.577 kcal/d (*p* < 0.05) and increase physical activity probability by 2.2% (*p* < 0.1), while children with a light-body cognitive bias, calorie intake increases by 43.557 kcal/d (*p* < 0.1) and physical activity probability by 3.2% (*p* < 0.05). Body cognitive biases affect children’s “eating-activity balance” and leads to body type deviation. We provide direct evidence, confirming that both calorie intake and expenditure influence the deviation from a healthy body shape caused by body cognitive biases.

**Table 11:** Body type deviation mechanisms for two types of body cognitive bias.

Variables	Calorie intake		Calorie consumption	
	(1)	(2)	(3)	(4)
Heavy-body cognitive bias	−37.577**		0.022*	
	(−2.239)		(1.834)	
Light-body cognitive bias		43.557*		−0.032**
		(1.846)		(−1.978)
Control variables	Yes	Yes	Yes	Yes
Year fixed effects	Yes	Yes	Yes	Yes
Provincial fixed effects	Yes	Yes	Yes	Yes
Constant term	872.900***	825.058***		
	(6.674)	(6.372)		
*N*	4,166	4,166	3,940	3,940

*p < 0.10, **p < 0.05, ***p < 0.01. The first two columns in parentheses are *t*-values under robust standard errors, and the last two columns are *z*-values.

## 6 Discussion and conclusion

### 6.1 Discussion

This article constitutes the first scholarly investigation into peer effects and health consequences of various body cognitive biases among Chinese children. Body cognitive biases are common among Chinese children, but existing research and policy formulation have not paid enough attention. We have confirmed both light- and heavy-body cognitive biases have peer effects. This support previous research showing that accepting incorrect weight evaluations from peers can directly affect a person’s weight development (24). We find that the peer effect is greater in adolescent groups, which is consistent with previous studies showing that adolescents are more susceptible to the influence of perceived peer weight norms (26). Furthermore, our study find the peer effect of body cognitive bias significantly affects children in rural communities, which offer a novel perspective on the documented double-burden of malnutrition faced by rural children in China (52). This corroborates existing literature indicating that unequal health opportunities are intrinsically linked to children’ environment (53). Besides, we find that the peer effect of light-body cognitive bias is stronger in girls, while the peer effect of heavy-body cognitive bias is stronger in boys, which once again supports the Gender Schema Theory (40). Previous studies have noted gender disparities in obesity rates among Chinese children (54), our finding suggests that a stronger peer effect of light-body size cognitive bias on boys may be a contributing factor. Finally, we find both light- and heavy-body cognitive biases contribute to deviations from a healthy body shape. The former predisposes children towards obesity, while the latter towards emaciation. Notably, both biases impact calorie intake and consumption. Given the habit formation effect (55), this portends a risk of exacerbated health deterioration in the future and impedes the execution of various health strategies in China.

Limitations include: data constraints prevent us from examining the underlying mechanism of peer effects of two body cognitive biases. Furthermore, while we do confirm the distinct effects of various types of body cognitive biases on children’s physical health, it remains unknown whether these biases will have long-term consequences on health status, human capital, and socio-economic standing. Additionally, previous studies have identified an intergenerational transmission effect in mental health (56), which raise the question of whether body cognitive biases may also propagate through generations. This is a potential research topic that warrants future inquiry. Moreover, the dataset used in this article did not cover recent data on cognitive biases in Chinese children. Future research will aim to collect updated data and further discuss the blocking mechanisms of peer effects on children’s body cognitive biases.

### 6.2 Conclusion

Both the light-body cognitive bias and the heavy-body cognitive bias have peer effects in Chinese children, which vary by age, gender, and community characteristics. The former causes children to become overweight by increasing calorie intake and reducing calorie expenditure, while the latter has the opposite effect.

The findings have relevant policy implications: (1) Society should be vigilant and promptly implement effective measures to understand the extent and severity of body cognitive biases among Chinese children; (2) Health, education, and media agencies should collaborate to develop preventive strategies, increase publicity on the risks of body cognitive biases and the benefits of healthy posture, enhance public awareness, and effectively use peer influence to counter unhealthy body image ideologies; (3) Expand the prevention path for overweight, obesity, and malnutrition in Chinese children from the perspective of body cognitive biases.

## Data availability statement

Publicly available datasets were analyzed in this study. This data can be found here: https://www.cpc.unc.edu/projects/china .

## Author contributions

YZ: Conceptualization, Data curation, Software, Writing – original draft. XW: Data curation, Software, Writing – review & editing. WZ: Writing – review & editing.

## Footnotes

^1^For policy documents, please visit the website below: https://www.gov.cn/zhengce/zhengceku/2020-10/24/content_5553848.htm; http://www.scio.gov.cn/ztk/xwfb/46/11/Document/976030/976030_1.htm

^2^ https://www.cpc.unc.edu/projects/china/about/proj_desc/survey

^3^Details for model explanation: https://www.stata.com/manuals/rivprobit.pdf

^4^ https://www.cpc.unc.edu/projects/china/data/questionnaires/

^5^ http://dg.cnsoc.org/article/2016b.html

^6^It aligns with Chinese screening norms for malnutrition and for overweight/obesity in children: http://www.nhc.gov.cn/wjw/pqt/201407/ffa.shtml, http://www.nhc.gov.cn/wjw/pqt/201803/a7962d1ac01647b9837110bfd2d69b26.shtml

^7^Detailed information about controlling variables is at: https://www.cpc.unc.edu/projects/china/data/questionnaires/

^8^ http://www.who.int/topics/adolescent_health/en/
